# Using natural travel paths to infer and compare primate cognition in the wild

**DOI:** 10.1016/j.isci.2021.102343

**Published:** 2021-04-15

**Authors:** Karline R.L. Janmaat, Miguel de Guinea, Julien Collet, Richard W. Byrne, Benjamin Robira, Emiel van Loon, Haneul Jang, Dora Biro, Gabriel Ramos-Fernández, Cody Ross, Andrea Presotto, Matthias Allritz, Shauhin Alavi, Sarie Van Belle

**Affiliations:** 1 Evolutionary and Population Biology, Institute for Biodiversity and Ecosystem Dynamics, University of Amsterdam, Amsterdam, the Netherlands; 2 Department of Cognitive Psychology, Faculty of Social Sciences, Leiden University, Leiden, the Netherlands; 3 ARTIS Amsterdam Royal zoo, Amsterdam, the Netherlands; 4 Department of Social Sciences, Oxford Brookes University, Oxford, UK; 5 Oxford Navigation Group, Department of Zoology, Oxford University, Oxford, UK; 6 Centre for Social Learning and Cognitive Evolution, School of Psychology and Neuroscience, University of St Andrews, St Andrew, UK; 7 Scottish Primate Research Group, Scotland, UK; 8 Centre d’Écologie Fonctionnelle et Évolutive, Université de Montpellier, Montpellier, France; 9 Eco-anthropologie, Muséum National d’Histoire Naturelle, CNRS, Université de Paris, Paris, France; 10 Theoretical and Computational Ecology, Institute for Biodiversity and Ecosystem Dynamics, University of Amsterdam, Amsterdam, the Netherlands; 11 Department of Human Behavior, Ecology and Culture, Max Planck Institute for Evolutionary Anthropology, Leipzig, Germany; 12 Department of Brain and Cognitive Sciences, University of Rochester, Rochester, USA; 13 Department of Mathematical Modelling of Social Systems, Institute for Research on Applied Mathematics and Systems, Universidad Nacional Autónoma de México, Mexico City, Mexico; 14 Center for Complexity Sciences, Universidad Nacional Autónoma de México, Mexico City, Mexico; 15 Department of Geography and Geosciences, Salisbury University, Salisbury, MA, USA; 16 School of Psychology and Neuroscience, University of St Andrews, Scotland, UK; 17 Department for the Ecology of Animal Societies, Max Planck Institute of Animal Behaviour, Konstanz, Germany; 18 Centre for the Advanced Study of Collective Behaviour, University of Konstanz, Konstanz, Germany; 19 Department of Biology, University of Konstanz, Konstanz, Germany; 20 Department of Anthropology, University of Austin at Texas, Austin, TX, USA

**Keywords:** Biological Sciences, Neuroscience, Cognitive Neuroscience

## Abstract

Within comparative psychology, the evolution of animal cognition is typically studied either by comparing indirect measures of cognitive abilities (e.g., relative brain size) across many species or by conducting batteries of decision-making experiments among (typically) a few captive species. Here, we propose a third, complementary approach: inferring and comparing cognitive abilities through observational field records of natural information gradients and the associated variation in decision-making outcomes, using the ranging behavior of wild animals. To demonstrate the feasibility of our proposal, we present the results of a global survey assessing the availability of long-term ranging data sets from wild primates and the willingness of primatologists to share such data. We explore three ways in which such ranging data, with or without the associated behavioral and ecological data often collected by primatologists, might be used to infer and compare spatial cognition. Finally, we suggest how ecological complexity may be best incorporated into comparative analyses.

## Highlights

• Comparing animal ranging decisions in natural habitats has untapped potential
• How decisions vary with natural information gradients reveals wild animal cognition
• Ranging data on at least 164 populations of 105 wild primate species are available
• We present three thought analyses to compare cognition and explain its evolution

## Graphical abstract

**Figure:**
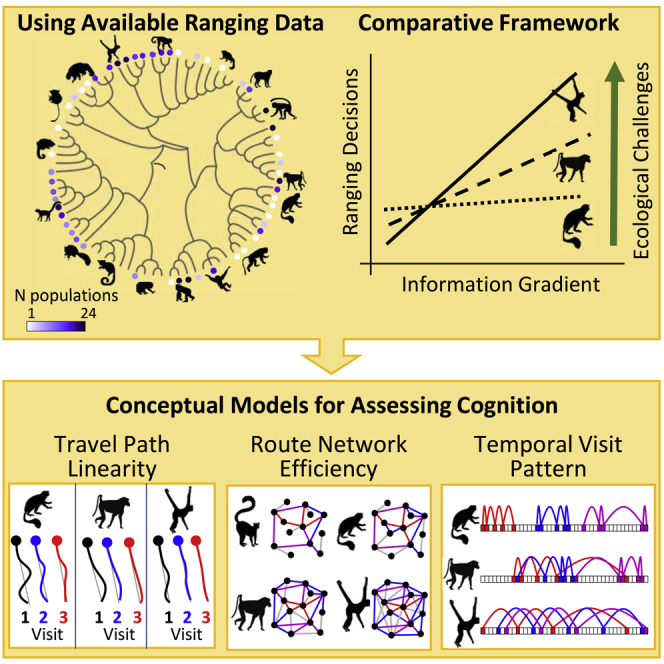

## Introduction

Comparative analyses are fundamental to understanding the evolution and development of cognition (Harvey and Krebs, 1990; Nunn, 2011; Romanes, 1884). Given the definition of cognition as a set of mechanisms that enable individuals to acquire, store, integrate, and act upon information (Shettleworth, 2010), decision-making tasks represent a useful window into cognitive abilities. Most studies of animal cognition are experimental and conducted on captive subjects (e.g., see Janmaat, 2019 for a review focused on primates; Morand-Ferron et al., 2016). These studies often include decision-making experiments designed for cross-species comparisons (e.g., ManyPrimates et al., 2019a). Captive experimentation enables control over potentially confounding factors and allows researchers to distinguish between alternative cognitive hypotheses (Jacobs and Menzel, 2014).

Exclusive reliance on lab-based studies, however, has downsides. First, the socio-ecological relevance of experimental cognitive tasks and the cognitive abilities observed may not generalize well outside of the experimental context (Calisi and Bentley, 2009). Second, experimental approaches generally restrict the number of species, populations, and developmental conditions available for comparative study (ManyPrimates et al., 2019b). Third, exclusive reliance on captive studies limits our understanding of local adaptations in cognition (Croston et al., 2015) and risks focusing on individuals with cognitive specializations not representative of the species as a whole, due to differences in challenges across development (e.g., a lack of foraging in large-scale space, escaping predators, or interaction with neighboring groups) (Janmaat, 2019; Ricklefs and Wikelski, 2002). Captivity-imposed restrictions on movement can give rise to diminished navigation performances (Jacobs and Menzel, 2014; Janmaat, 2019; Menzel and Beck, 2000), and there is evidence of important effects that socio-ecological environments have on even basic cognitive mechanisms, from the neural (e.g., Bogart et al., 2014; Henrich et al., 2010) to the behavioral level (e.g., Haun et al., 2011). To gain further insights into the development, plasticity, and evolutionary drivers of cognition, it is therefore essential to extend the taxonomic coverage of our study species and to also study populations within their natural habitats.

An alternative approach to studying cognition uses proxy variables for cognitive abilities that can be acquired without having to observe animals in situ. These proxies typically consist of brain measurements (e.g., hippocampus size, neo-cortex ratios, relative or absolute brain volume, or neuron counts; Barks et al., 2015; Barton, 1996; DeCasien et al., 2017). Such studies then attempt to relate these variables to predictor variables measuring socio-ecological challenges, via comparative phylogenetic methods (Byrne, 1996). Nonetheless, conclusions are limited by uncertainty about the functional relationship between brain measures and cognitive abilities (Chittka and Niven, 2009; Healy and Rowe, 2007; Isler and van Schaik, 2006; Logan et al., 2018).

Here, we propose a third, complementary approach: studying “decision-making in wild animal populations using unmanipulated field observations”. Specifically, our aim is to develop ways of measuring and comparing indicators of cognition that (1) are naturally exhibited by animals, (2) require no experimental manipulation, and (3) can be collected at all field sites, allowing large-scale comparisons. We focus on animal ranging behavior (i.e., Global Positioning System [GPS] tracks) because large amounts of such data are available (Cagnacci et al., 2010; Kays et al., 2015; Nathan et al., 2008; Tomkiewicz et al., 2010; Williams et al., 2020). Ranging behavior is key to many biological imperatives influencing natural selection (including acquisition and defense of food, locating mates and shelter, and avoidance of predators and competitors) and involves numerous distinct components and types of cognition (e.g., procedural, declarative memory [e.g., episodic memory], categorization, intuitive statistics, serial learning, spatial reasoning, and future planning; see Glossary) (Fagan et al., 2013; Janmaat et al., 2016; Trapanese et al., 2019).

**Glossary**

1 **Cognition**: mechanisms by which animals acquire, process, store, and act on information from the environment.
2 **Navigation**: the cognitive process involved in moving from one place to another.
3 **View matching** (also called view-dependent place recognition or landmark-based image matching): a method that guides movement decisions by comparing the current view with a template, which is a snapshot memory of the visual panorama surrounding a goal location. The animal moves such as to minimize the discrepancy between the current retinal image and the template. Matching may include multiple consecutive images, incorporating how sizes, edges or angles in a scene are expected to change upon approach, or multiple templates from different vantage points for the same goal.
4 **Path integration** (also called dead reckoning, a corruption of “deduced reckoning”): a method of keeping track of one’s current location relative to a known ‘home’ location by adding vectors of the separate effects of each sector of travel. It requires repeated estimations of speed, time and direction, and without instruments errors from these estimated vectors accumulate reducing accuracy. Path integration yields a travel vector from the ‘home’ location; reversing the vector allows direct return travel in a beeline, potentially aided with intimate knowledge of a small area around the home base to correct for any errors from path integration (e.g., by means of view matching).
5 **Procedural memory** (also called implicit memory, unconscious memory, or automatic memory): a category of long-term memory that stores information on how to perform certain procedures (e.g., the procedure of brachiating, go straight and turn right at the tree), and aids in performing certain tasks without the need for conscious control or attention.
6 **Declarative memory** (also called explicit memory): a category of long-term memory that refers to making a mental representation of experiences, events, facts, and information used in everyday living. As opposed to procedural knowledge, which is “knowing what to do”, declarative memory refers to “knowing what is there” (e.g., that the goal is located 100 m to the east of the tree). It may include both semantic memory (storage of factual knowledge independent of personal experience) and episodic memory (storage of personal experiences, and events in life).
7 **Episodic memory**: the memory system that encodes, stores, and retrieves a unique, personal experience associated with a precise spatial and temporal context. It supports an individual’s ability to consciously re-experience past events and possibly to pre-experience future events. The content of episodic memory is composed of information regarding *what*, *where* and *when* a specific event occurred.
8 **Spatio-temporal memory**: umbrella term for memory processes that involve encoding, storing and retrieving the location of objects or places in the environment and/or temporal patterns of specific resources (e.g., fruit production patterns of trees, caches expiration rate). The spatial (*where*) and temporal (*when*) elements of memory can be stored independently from each other or in combination. Re-experiencing the original encoding of the memory (= episodic memory) does not need to be involved in retrieval.
9 **Serial learning**: the learning of a sequence of stimuli, items, locations, or responses, allowing the individual to recall or execute the same set of items or responses in a specific order. In the context of animal movement, serial learning may be relevant to navigation by memorising a specific chain of landmarks or locations visited, a specific sequence of movement vectors and directional shifts, or both.
10 **Intuitive statistics:** the ability to make inferences about the frequency of a trait in a population (e.g., the number of fruit-bearing trees within a tree species). This inference can be from samples to population, e.g., inferring, from seeing a few individual trees, an expectation about the general frequency of trees of the same species that are currently bearing fruit; or the inference can be population to sample, inferring, from seeing a number of trees, an expectation that a specific individual tree is also bearing fruit. This ability can, for example, enable primates to keep track of proportions of fruit-bearing trees within tree species (e.g., species with high synchrony in fruiting where all individual trees fruit simultaneously), irrespective of their density.
11 **Future Planning**: the ability to act for a future motivational state - an ability to perform a combination of unique actions (e.g., nest positioning and breakfasting), with the time of the planning action (e.g., nest positioning) being separated from the future activity (e.g., breakfasting) by a period of other activities, (e.g., climbing, nest building, and sleeping). Note that planning on its own can be defined as: The ability to act in accordance with a future goal, independent of its current motivation.
12 **Heuristic**: a simple and efficient rule employed in problem solving that is not necessarily an optimal approach yet yields a satisfactory solution. Examples are trial-and-error, rules of thumb, and educated guesses, and can decrease the cognitive load associated with making decisions.
13 **Constancy**: a component of predictability based on information theory; it is the reverse of variability. As an example, if the productivity of a patch has a high value of constancy, a primate sampling it at a given time may gain information on its future state. In contrast, low values imply that sampling such patch, in the absence of any other variable, does not provide information of the future productivity of that patch.
14 **Contingency**: another component of predictability based on information theory, quantifying the degree to which one variable is statistically dependent on another variable. For example, if the fruiting time series of two plant species is statistically dependent on one another, perhaps with a time lag, then a primate sampling the fruiting state of one species may gain information on the present and/or future fruiting state of the other species.
15 **Sensory gradient**: rate of regular ascent or descent of cues emitted by a target (e.g., a food source) that can be perceived by the olfactory, auditory or visual senses of an animal that travels towards that target.

## Cognition in ranging behavior

Field researchers routinely record the movement of their study individuals or groups, accumulating such data over decades. Moreover, recent years have seen enormous advances in bio-logging technology, yielding large longitudinal data sets (Cagnacci et al., 2010; Kays et al., 2015; Tomkiewicz et al., 2010; Williams et al., 2020). Such data sets offer a potentially powerful opportunity to infer cognition from travel data (Fagan et al., 2013; Janmaat, 2019; Nathan et al., 2008; Ross et al., 2018). However, to date, relatively few movement ecology papers have addressed cognitive questions (e.g., Fagan et al., 2013; Joo et al., 2020; Pacheco-Cobos et al., 2019).

Inferring cognition from movement data is not straightforward. Multiple factors can explain where, when, and how an animal travels (Codling et al., 2008; Janson and Byrne, 2007; Nathan et al., 2008). For instance, travel paths can be influenced by locomotor and energetic constraints, the heterogeneous distribution of resources in the landscape, and/or the animal's internal motivation (Nathan et al., 2008; Wilson et al., 2013). Moreover, an observed movement pattern can often be explained by several cognitive hypotheses that are difficult to tease apart. For example, straightline travel could arise from traveling along a sensory gradient, path integration, group inertia, or navigation based on use of direct spatial memory (a map-like mental representation of a given area), see Glossary (Nathan et al., 2008). Similarly, the same cognitive adaptation can be expressed through different types of behavior; for example, memories of key points of interest may give rise to (1) faster travel, (2) straighter travel, (3) changes in travel direction, (4) longer approach distances, (5) frequent revisits, and/or (6) non-random sequential orders of revisits to important locations (Table 1). Because ranging data usually allow for measurement of each of these indicator variables, such suites of behavioral indicators can be used to our advantage to test more robustly the underlying cognitive processes.

**Table 1:** Examples of movement decision outputs using x,y,t-data from animals in their natural habitat

Spatial scales of movement decision outputs	Movement decision metric derived from GPS data	Example	Inferred mechanisms		Potential effect on efficiency/fitness
			Cognitive mechanisms that enable mental representation	Other mechanisms (rules of thumb/heuristics/perception)	
Travel bout or a single trajectory between two locations	Shape	Lowland gorillas' travel paths increase linearity when high value food rewards are found at target locations (Salmi et al., 2020).	Use long-term spatial memory of food resource location, quantity, and quality when traveling.	Beaconing to sensory cues (e.g., fruit scent or conspecific or hetero-specifics acoustic cues), landscape attributes (e.g., forest openness, inter-tree connectivity), avoid backtracking.	Linear paths minimize time and energy spent on traveling to target.
	Speed	Capuchin monkeys increase their travel speed toward trees with fruit, according to the expected reward (Tujague and Janson, 2017).	Track phenological patterns of fruit trees and anticipate the emergence of fruit amounts and competition levels within the group at specific trees.	Beaconing to sensory cues was discarded in the study by non-linear effect of fruit amount on speed: speed increased at low values but decreased again at very high values when olfactory cues are strongest.	Higher speed when approaching a destination gives access to valuable resources more quickly and ahead of competitors.
	Approach distance	Mantled howler monkeys travel long distances through their home range to reach highly valuable food sources, forgoing other food sources at shorter distances (Hopkins, 2016).	Integrate long-term spatial memory of resource distributions to choose destinations that maximize resource gain, exhibit self-control.	Travel linearly in a random direction, stop at first food patch that exceeds criterion value (lower criterion if hungry).	Trading off the energetic cost of longer travel against greater reward can improve reward rate compared to immediate smaller rewards.
	Departure time	Chimpanzees depart from their nests in the morning earlier when their planned “breakfast” site (i.e., fruit tree) is sought after and far away (Janmaat et al., 2014).	Use long-term memory of multiple locations and travel distance as well as ephemerality levels of food types, flexibly plan travel time, exhibit conditional decision-making.	Learning of time-place associations was discarded in the study as chimpanzees built a nest at different locations each night.	Reaching fruit trees in the early morning before competitor likely enhances chimpanzees' foraging efficiency.
Daily path	Shape (e.g., change points)	White handed gibbons' significant directional changes are located at preferred fruit trees that are ripening (Asensio et al., 2011).	Use spatial memory of location of preferred fruit trees and plan direct travel bouts between them.	Beaconing to sensory cues or landscape attributes surrounding the fruit trees produce goal-directed movement.	Iterative goal-directed movement toward locations where biologically meaningful events are likely to occur (e.g., fruit ripening) increases likelihood of reaching these at optimum times.
	Order of visits	Bumble bees optimize the order in which nectar flowers are visited with experience based on the distance between resources and the production of pollen and nectar in each flower (Lihoreau et al., 2010).	Use long-term memory of multiple locations and travel distance as well as food amount, compute shortest path connecting a set of flowers, exhibit route planning.	Beaconing to sensory cues, random search, nearest neighbor rule (e.g., move to nearest feeding site consistently), convex hull strategy (e.g., approach feeding sites based on their distance to the edge of the feeding area).	Minimizing overall distance traveled between destinations saves time and energy, maximizes reward rate.
Weekly, monthly, annual, multi-annual paths	Revisits	Mangabey monkeys avoid revisiting trees they depleted during previous visits and monitor those they did not feed in yet (Janmaat et al., 2006).	Use long-term spatial memory of recent feeding events and their locations.	Beaconing to sensory cues was discarded in the study as both tree types did not bear fruit, random search was discarded as revisiting patterns were not random.	Avoiding previously visited and depleted feeding sites saves time and energy.
	Path recursion/overlap	African elephants repeatedly reuse paths in the periphery of their home range but engage in more flexible movements within the core area (Presotto et al., 2019).	Use a vector (coordinate-based) or network map in familiar areas while relying on landmarks and memorized routes outside of familiar areas.	React to landscape attributes (e.g., steep slopes and availability of substrate to navigate) that can constrain movement in the periphery.	Reuse of known paths minimizes energetic cost of travel in heterogeneous/unfamiliar landscape; flexible movement allows shorter travel between targets and fosters exploration for new resources.
	Path networks	Bees travel between flowers more optimally with increasing experience but only at large spatial scales when the cost of non-optimal foraging behavior is thought to be higher (Pasquaretta et al., 2017).	Use a vector (coordinate-based) or network map of the large-scale resource distribution, compute approximate optimal path connecting set of targets at large spatial scales.	Beaconing to sensory (panoramic visual) cues when navigating in large-scale space but not in small scale space. Using information of distant valuable locations from others in the nest (waggle dance).	Minimizing overall distance traveled between destinations saves time and energy, maximizes reward rate.

Also shown are inferred cognitive mechanisms as potential explanations and the hypothesized effect of these outputs on efficiency/fitness.

Related literature: (1) travel bout [shape: Jang et al., 2019; Janmaat et al., 2006; Normand and Boesch, 2009; Toledo et al., 2020; speed: Jang et al., 2019; Janmaat et al., 2006; Noser and Byrne, 2007; Tujague and Janson, 2017; approach distance: Ban et al., 2014; Polansky et al., 2015; departure time: Abrahms et al., 2019; Bracis and Mueller, 2017]; (2) daily path [shape: Byrne et al., 2009; de Guinea et al., 2021; Janmaat et al., 2013; order of visits: Lihoreau et al., 2010; Riotte-Lambert et al., 2017; Teichroeb, 2015]; and (3) weekly, monthly and annual paths [revisits: Berger-Tal and Bar-David, 2015; Bracis et al., 2018; Janmaat et al., 2013; path recursion/overlap: Bracis and Mueller, 2017; de Guinea et al., 2019; Di Fiore and Suarez, 2007; Presotto et al., 2019; Presotto and Izar, 2010; path networks: Green et al., 2020b; Latty et al., 2011; Pasquaretta et al., 2020].

In this paper, we will focus on primate ranging data, although our methodology is relevant to other taxa as well (e.g., Collet and Weimerskirch, 2020). We focus on primates for several reasons: (1) the primate order contains at least 505 species (Rowe and Myers, 2016) and is well-suited for comparative analyses, which require large data sets with known phylogenetic branch lengths (Arnold et al., 2010), (2) most primates are diurnal and can be followed for the entire period of their activity, enabling identification of motivations and goal-directed actions (e.g., Byrne et al., 2009), (3) high levels of site fidelity allow researchers to relocate individuals in subsequent years and thus enable longitudinal studies under changing socio-ecological conditions (e.g., Janmaat et al., 2009; Van Belle et al., 2018), and (4) although studies of cognition in wild primates are growing in frequency (Trapanese et al., 2019; Zuberbühler and Janmaat, 2010), there is an urgency to this work, as key habitats are rapidly disappearing, and with them the opportunities to study the development and evolution of primate cognition (Estrada et al., 2017; Junker et al., 2020).

The rest of the paper runs as follows: we first assess the availability and extent of primate ranging data using a global survey. Then, we discuss how cognitive abilities can be studied using the decision-making outcomes recorded in the movement patterns of wild primates. We present three conceptual approaches to analyzing such ranging data. We conclude with a discussion of how estimates of ecological complexity can be improved and incorporated into our proposed comparative analyses.

## Survey on availability of primate ranging data

**Figure 1:**
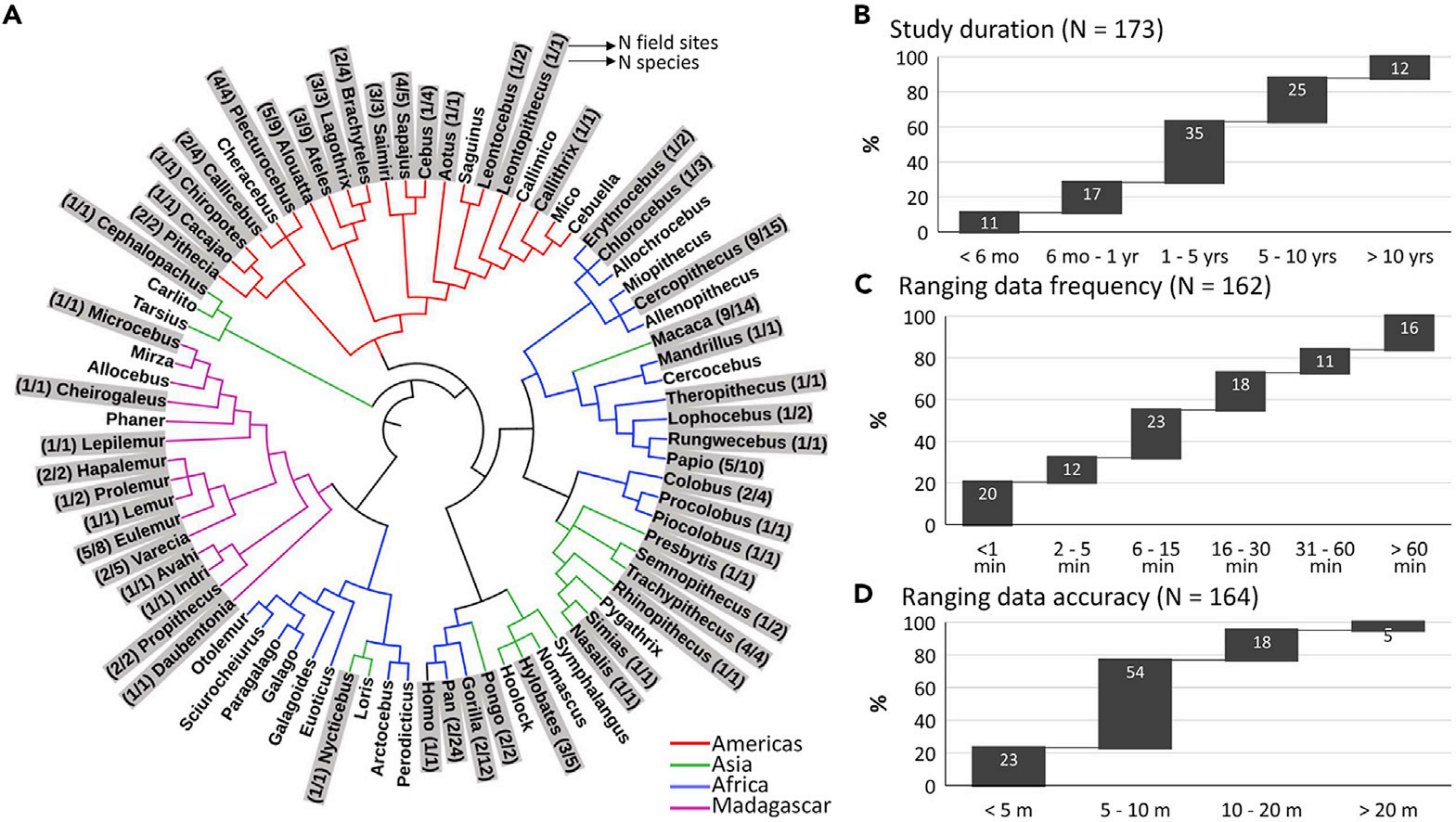
Results from the global survey (A–D) (A) Cladogram showing the primate genera represented in the survey (gray); percentages of studies according to (B) their duration, (C) the frequency with which GPS waypoints were recorded, and (D) the accuracy of the GPS unit(s) at the field site.

We invited over 300 primatologists to fill in a survey about ranging data for each of their field sites and study species. Although our survey does not represent an exhaustive list of field researchers, it appears nonetheless largely representative of the variation in the field. In the survey, 177 entries from 123 primatologists indicate that ranging data have been collected as part of their field protocols. The data represent at least 105 species (Figure 1A) at 120 field sites, representing 164 primate populations in 36 countries.

Most studies (72%) lasted >1 year (Figure 1B). Slightly over half (55%) collected ranging data relatively frequently (at ≤ 15-min intervals, Figure 1C), and these data were accurate to at least a 10 m radius in 77% of the studies (Figure 1D). In addition, respondents also indicated that they collected data on the study subjects' behavior (83%), food tree locations (69%), sleep tree locations (64%), food availability (55%), and nutritional values of food items (23%). Topographic information is freely available online via open-source repositories, but 31% of the participants reported having high resolution topographical information of their study area. Laser Imaging Detection and Ranging (LiDAR) information on forest structure is available in only 6% of the cases.

Only 10% of the respondents indicated that they had published all aspects they had planned pertaining to their ranging data. Many respondents had either published part (42%) or none (48%), respectively. Clearly there is a wealth of ranging data that have not been made available through publications. Furthermore, 89% of respondents claimed that they would be willing to share their ranging data (only 9% said that they would like to publish prior to data sharing, and only 2% of respondents said that they had reservations toward sharing their data).

## The comparative approach: Linking decision-making outcomes to variation in information gradients in natural habitats

**Figure 2:**
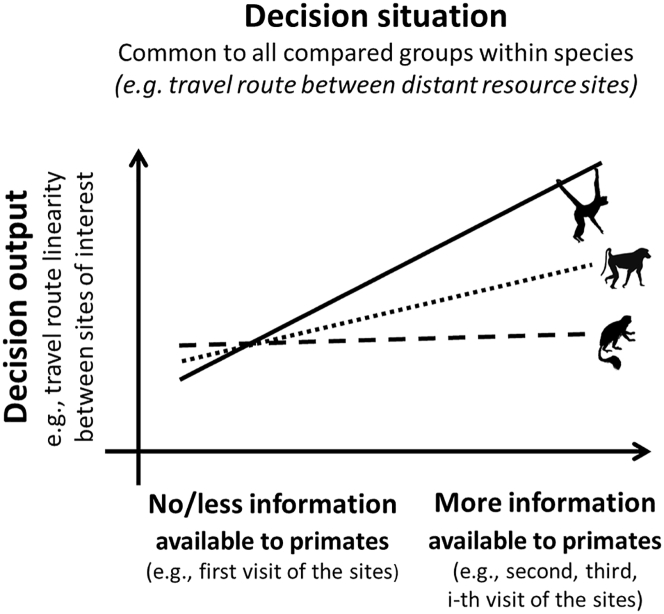
Graphic representation of the comparative framework Cognition can be studied in each species or population in the wild by comparing behavioral decision outputs (e.g., travel path linearity) when primates are naturally provided with different input of information (e.g., ranging within familiar vs less familiar areas). In this hypothetical example, we expect a spider monkey and baboon group (solid line and dotted line, respectively; slopes >0) but not a colobus group (dashed line; slope ∼0) to increase travel linearity with increasing familiarity with the area, suggesting more effective spatial learning in the first two groups but not in the latter.

In controlled experiments, cognition can be empirically studied by manipulating the amount of information provided to study subjects and subsequently examining how it affects their behavioral outcomes (Shettleworth, 2010). For our comparative approach, we propose examining the correlation between a measure of the variation in information available to wild animals (the “information gradient”) and the ranging decisions they make in their natural habitats. For example, an increasing number of visits to a specific area should increase an animal's experience with that habitat and thus give an indirect measure of acquired information. We can then measure the characteristics of a travel path (a “decision output”) to assess whether travel paths between key points of interest within a habitat become more efficient (e.g., more linear) with increasing information/experience. The resulting regression slope between these two variables (e.g., past visits and path linearity) then gives insight into the cognitive process of the animal (Figure 2). For example, with repeated visits, animals are expected to learn through repeated trial and error and navigate to rewarding locations more efficiently as memories of locations, route directions, and intermediate landmarks become more salient and accurate. When individuals revisit the area, this ever more precise information can become available to spatial cognitive processes like view matching and path integration, leading to further increases in path linearity. Additional examples of cognitively relevant decision outputs and natural gradients in information available to animals are provided in Tables 1 and 2. Table 1 provides a detailed overview of what potential cognitive processes can be inferred, ranging from making cognitive representations to using rules of thumb. For decision outputs, we focus on movement decisions, but the same idea could be applied to other behavioral measures. Likewise, we focus on information accumulated through experience, but measurements of empirical environmental gradients such as a gradient of fruit odor can also be analyzed (e.g., see our third example in the last row of Table 2).

**Table 2:** Examples of natural gradients in information available to animals making movement decisions, which can be measured to study cognition in the wild

Information gradient associated with variation in …	Quantification of information gradient	Related literature
… How many times a place has been previously visited	Number of previous visits recorded at location	Janmaat et al. (2013)
How long animals have been ranging in the area	Time elapsed since first records of presence in the area	Isbell et al. (1990, 1993)
Age or experience of group members	Mean or maximum age of group members	Teitelbaum et al. (2016)
How long ago a location was last visited	Time since last visit	Benhamou and Riotte-Lambert (2012); Watanuki et al. (2003)
Regularity of previous visits (clustered or regularly spaced in time)	Variance in delay between past consecutive visits to location	Henderson et al. (2006)
Sensory access to cues or goals (visibility from topography, vegetation density, weather; wind direction; etc.)	Distance/direction to nearest cue relative to estimated max perception distance/direction	Janmaat et al. (2006, 2013); Noser and Byrne (2007)

Standardizing the measures of information and behavioral output across different individuals, social groups, populations, or species will enable us to compare cognitive performances and processes (Figure 2). The next step is then to investigate the evolutionary drivers of differences in cognitive processes by designing multivariate statistical models that integrate socio-ecological complexity variables, potential confounding factors, and phylogenetic controls. In the following three sections, we detail three examples of such models as “in-concept” approaches to investigate the evolution and development of cognitive abilities.

## Example 1: Linearity of travel

When a primate makes a beeline from one resource to another over distances well exceeding its line of sight, it might seem obvious that it has used spatial knowledge to compute an efficient path, but there are many other possibilities (Byrne, 2000). Perhaps, although the resource itself is only visible at close range, it may be reliably associated with long-distance cues: a small waterhole, for instance, may be detected at long range by tall trees that only grow there; fruit-eating birds, such as hornbills or toucans, can visually scan vast areas of forest to detect trees with ripe fruit, and their subsequent flight paths and calls may be used as cues by primates; a whole tree of ripe fruit may even emit a scent plume that primates can simply home in upon. Even where subtle cues can be ruled out, traveling on a linear path may not reflect optimal efficiency (de Guinea et al., 2019; Guilford and Biro, 2014; Jang et al., 2019). Least-effort paths will depend on environmental features: slope angles, obstruction by cliffs or vegetation, and diversions to exploit foraging opportunities along the way. And in the absence of any spatial knowledge, highly linear travel (a correlated random walk; Ross et al., 2018) between resource patches is a good heuristic strategy that avoids wasteful backtracking. As such, since it makes sense to slow or stop when a valuable resource is encountered, observations of beeline travel “to” a resource may result even when no spatial knowledge was involved in the decision-making process.

**Figure 3:**
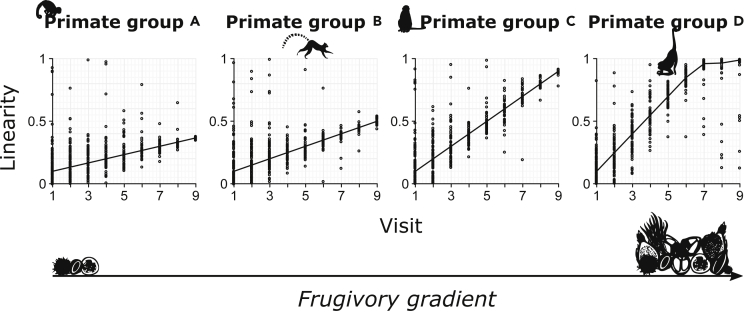
Illustration of a design for a comparative analysis of spatial performance Each data point represents linearity values (decision output) of a travel path at the x^th^ number of visits to the same end location (the information gradient). We predict a correlation between the regression slopes and a descriptor of an ecological challenge in the primate group's environment, such as percentage of fruit, or the average ephemerality level of the fruit species in the diet. Pairs of given start and end locations should be included as a random effect in the regression model. The presented data are hypothetical.

These difficulties mean that studies successfully using travel paths to index primate knowledge of large-scale space have been restricted to those with very detailed recording of individual animals' foraging histories (Janmaat et al., 2006), estimations of detection distances and elaborate survey data of nutritional and other resources over the entire home range (Jang et al., 2019; Janmaat et al., 2013), or others who simply grabbed lucky breaks, where opportune circumstances have set up a “natural experiment” in the field (Garber, 1989). Here, we propose an approach that can be applied more generally, even when only x,y,t-data (x and y coordinates plus time) are available: by analyzing “how primates improve their route linearity with experience”. If we observe that travel gradually increases in linearity, in less frequented areas, from the first to the last time an individual traveled between two points of interest, and we have reasonable evidence that the conditions of movement (e.g., obstacles, motivation) do not vary much between the first and last visits, then we can make a strong case for information use and spatial cognition. To test such predictions, hierarchical regression models can be used to test for a significant positive slope between linearity (the decision output) and experience (the information gradient). In principle, the rate of increase in linearity with experience is an index of spatial cognition that can be compared between different populations and species (Figure 3). To do so, confounding factors that are measurable across data sets (e.g., slope, distance to the goal, visibility of the goal, variance in approach direction) can be included as covariates, with additional controls for individual- or group-specific factors (random effects).

More generally, we can test how travel linearity between points of interest in large-scale space depends on the time elapsed between successive visits to compare the length of memory windows. In addition, we can measure effects of visit regularity (variance in time between previous successive visits), the duration of previous visits, and the profitability of previous visits. Comparison between the core range of a group and peripheral areas can also help: in highly familiar areas, we might expect spatial knowledge to be at a plateau, but in peripheral areas, repeated visits may increase movement efficiency. The factors needed to test such predictions are, in principle, extractable from pure x,y,t-data.

Finally, we can use this general approach to test specific comparative hypotheses about the evolution of cognition, by including predictor variables that are indicative of the challenges faced by each primate group. For instance, we might expect that learning would be more effective in species that necessarily confront higher ecological complexity. The percentage of fruit in the diet has often been used as a proxy for foraging complexity: we might expect the slope of the relationship between experience and travel linearity to be steeper in more frugivorous species (Figure 3).

In cases where only x,y,t-data are available (i.e., points of interest are not known from focal observation), the researcher can make use of significant change points in travel trajectories to identify key locations of interest (Asensio et al., 2011; Byrne et al., 2009; de Guinea et al., 2021; Janmaat et al., 2013). Results of such analyses can subsequently be compared to results gleaned from populations in which more detailed behavioral and ecological data from on-the-ground focal follows are available (e.g., food tree locations), in order to assess their robustness. Annotated data on group size could, in addition, distinguish individual knowledge from collective knowledge in fission-fusion societies or during dispersal activities.

For this approach to work, one needs to account for variation in how data were collected across sites and populations. To estimate familiarity, several methods are available (see Kie et al., 2010), but utilization distributions (location or movement-based kernel density estimates; Benhamou, 2011; Noonan et al., 2019) are now widely used to define animals' home ranges and estimate space usage based on x,y,t-data. Our approach can be similarly useful for exploring the effects of between-group and between-population variation in ecological variables within species.

## Example 2: Route network efficiency

Our second example concerns a comparative analysis of travel efficiency of different primate groups, where familiarity with different areas of the home range represents the information gradient. Within a home range, primates learn to travel efficiently toward many resource sites. To do so, individuals may calculate a novel path from their current location to the target or they may travel along previously used paths. The first option requires individuals to memorize and calculate distances and angles among many locations (Garber, 2000; Tolman, 1948) and has been reported in only few primate populations (chimpanzees, *Pan troglodytes*, Normand and Boesch, 2009; black capuchin monkeys, *Sapajus nigritus*, Presotto and Izar, 2010; humans, *Homo sapiens*, Haun et al., 2011). The latter option, considered ubiquitous among primate species (and other animals; Trapanese et al., 2019), is thought to be less cognitively demanding. Less information needs to be stored when restricting movements to a finite number of travel paths that are repeatedly traveled (de Guinea et al., 2019; Green et al., 2020a; Warren, 2019). Recurrent travel through the same areas allows individuals to generate a network of habitually used routes joined by “nodes”, locations where movement decisions take place (Garber, 2000; Perna and Latty, 2014; Presotto et al., 2018). Though less cognitively demanding, route networks can differ dramatically in complexity and efficiency across groups, populations, and species (Gallotti et al., 2016), even when controlling for the number of nodes and environmental attributes (Barrat et al., 2005; Perna and Latty, 2014).

We propose that comparing route network structure across different populations can provide powerful insights into their relative spatial competence: specifically, their ability to construct networks that optimize travel effort among a set of resource sites. To do so, we need standardized methods for determining the location of routes and nodes. The Habitual Route Analysis Method tool (HRAM; script available on Git-hub: https://github.com/apresotto/Habitual-Route-Analysis-Method) identifies habitual routes systematically (Curry and Presotto, 2018). The detection of nodes can be systematized by applying the change point test (CPT; Byrne et al., 2009) to the movement patterns of the study population. If significant directional changes are detected in the proximity of an intersection between habitual routes, we may conclude that the study population takes directional decisions at this location and, therefore, that the location can be considered as a node within the route network (Presotto et al., 2018).

**Figure 4:**
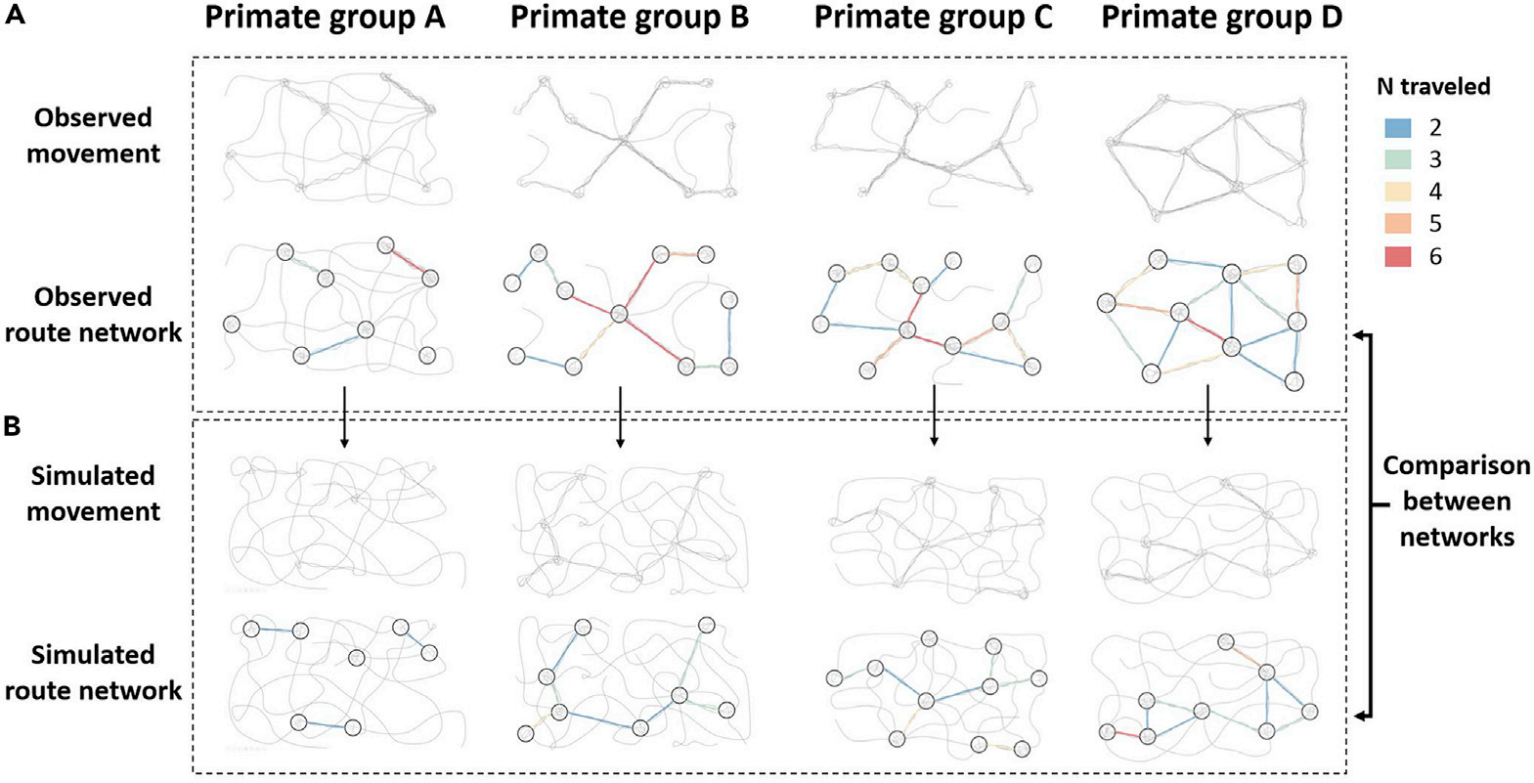
Graphical representation of route network efficiency (A) observed ranging patterns of four wild populations and the corresponding route networks described after applying the Habitual Route Analysis Method tool and change point test. (B) Simulated random ranging patterns based on metrics (e.g., step length, turning angle) derived from the observed movement patterns of each population. Colors indicate the number of times that the study populations traveled between a given pair of nodes. The presented data are hypothetical.

Route networks can then be compared across groups or populations at different, complementary levels to enhance our understanding of the variation in cognitive capacity (e.g., learning speed and long-term memory storage) across primate species. First, we can gain insight into the complexity of a route network by calculating the number of nodes and the links between them. Second, by characterizing the structure of networks as weighted graphs, we can infer the efficiency and complexity of these networks (Barthélemy, 2011). For instance, calculating how many times each node appears in the shortest path between other pairs of nodes (i.e., betweenness centrality) or how connected it is to other well connected nodes (i.e., eigenvector centrality) will indicate whether primates primarily navigate through a limited set of nodes to reach their goals or use a large number of nodes at comparable frequencies (Bast et al., 2016; Figure 4). Third, we can also quantify the information involved in making movement decisions using entropy-derived measures as proxies for the cognitive load faced by individuals (Gallotti et al., 2016). For instance, the more interconnected and complex a route network is, the more information will be required to compute movement decisions through it (Gallotti et al., 2016).

Route networks generated by wild primates are also influenced by landscape attributes and the spatial distribution of food resources (de Guinea et al., 2019; Presotto et al., 2018). To investigate the efficiency of an empirical route network on a given landscape, it is therefore important to compare the network structure of the observed movement patterns against those of simulated networks. Simulations based on random walk patterns (Suarez et al., 2014) can be conducted using the metrics of movement patterns (e.g., step length, turning angle, probability of behavioral shifts; Patterson et al., 2008) observed in a given species or population and can include a resistance layer that reflects the probability that study subjects move across different landscape attributes (e.g., steep slope; Quaglietta and Porto, 2019). We can then use HRAM and CPT to construct a route network and determine nodes for the simulated data (Figure 4) sets and subsequently compare the observed and simulated networks by the mean route factor (or detour index) to calculate a relative mean route factor. This is a common measure of efficiency based on the difference between the direct Euclidean distance between pairs of nodes and the shortest possible distance between them along routes within the route network (Barthélemy, 2011). We would predict that primate species with more flexible spatial cognitive abilities would show networks with lower mean route factors than those of simulated networks.

If route network complexity indeed varies with spatial cognition, the value of the relative mean route factor should decrease with experience in the landscape. To test this, we can design a hierarchical regression model that includes the difference between the mean observed route factor with the simulated one as a response variable and the familiarity of the primate group with different areas in home range (e.g., the core areas versus the periphery) as a predictor variable. Specific comparative hypotheses about the development and evolution of spatial cognition can be examined by adding socio-ecological descriptors of each primate group as covariates in the statistical model.

## Example 3: Constancy and contingency of revisit intervals

With many important food resources only available at scattered locations in the landscape and during limited time periods (Janmaat et al., 2016; Milton, 1981), cognitive abilities that enable a primate to know when to go where are expected to be crucial for efficient foraging. A food source may be depleted by competitors if an individual arrives late, but an early arrival can mean that energy is wasted in locomotion to a food source that is not yet consumable (Janmaat, 2019). Our third example concerns assessing primate species' abilities in acting upon spatiotemporal variation in food availability. Our approach builds on the concepts of “constancy” and “contingency”, introduced by Colwell (1974) to describe periodic and uncertain phenomena—mainly environmental variables—in nature. More recently, Riotte-Lambert and Matthiopoulos (2020) proposed that these concepts are wide-ranging constraints that dictate foraging strategies and survival across animal species.

**Figure 5:**
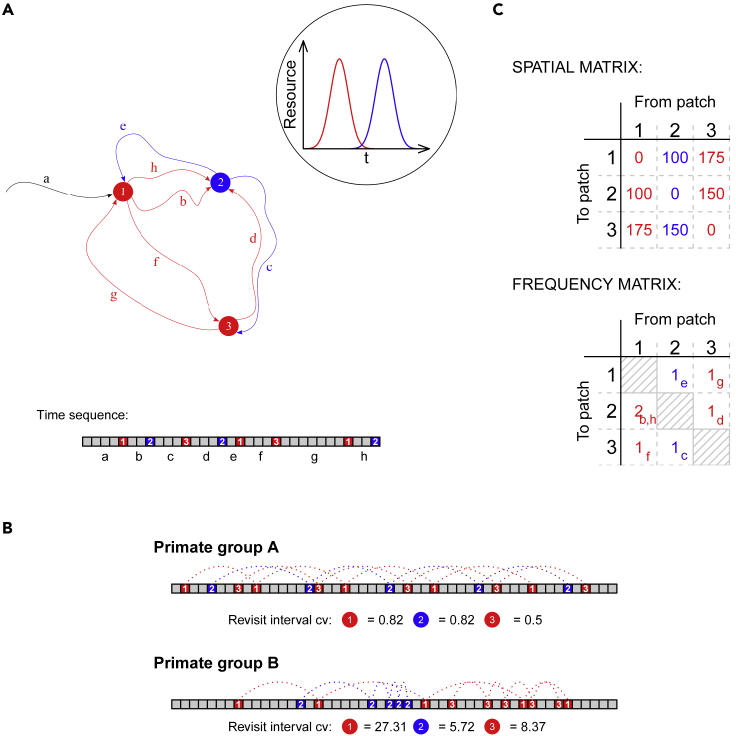
Illustration of a temporal pattern analysis using constancy and contingency to compare cognition (A) Schematic example of x,y,t-movement data translated into a travel network with locations of three food patches (identified by numbers) belonging to two species (identified by color) as nodes, interconnected by travel paths (identified by letters). The arcs depict the geographical location of the travel paths and are colored according to the patch where they originate. The time sequence below the travel network is subdivided into equal-duration segments, showing both the order of visiting the patches as well as the time spent within each patch. A segment during which a food patch was visited is colored according to the corresponding patch color, and the numbers corresponding with the different travel paths are shown below in the time sequence. In this example, each of the two species has its own fructification pattern with similar length but not overlapping in time. Both species fruit synchronously—here represented by the use of two Gaussian distributions with small variance. (B) Two distinct time sequences of food patch visits, illustrating revisit patterns associated with high regularity values (group A, upper sequence) and low regularity values (group B, lower sequence). Group A is characterized by regular revisit intervals and (corresponding) low coefficients of variation; group B shows the reverse: irregular revisit intervals and a high coefficient of variation. It is noteworthy that the constancy of revisit intervals may be different for different patches (as apparent in the second timeline) and can thus be analyzed at multiple levels to reveal more about the function of different patches. (C) Two matrices, summarizing the situation shown in the travel network in A. In the spatial matrix, each cell shows the geographical distances between two patches that were visited consecutively. In the frequency matrix, each cell shows the number of times two patches were visited consecutively; for example, the number 2 in the first column states that there are two travel paths that originate at patch 1 and lead to patch 2. This matrix can be treated as a contingency table, and the overall strength of association between the categories in the rows and columns can be expressed by Cramér's V.

Average patterns in resource visit schedules may provide clues about the use of learning and memory to model spatiotemporal variation. We propose two measures (decision outputs) to express this average pattern, corresponding to the environmental regularities in constancy and contingency: (1) “regularity” (the constancy in interval between revisits to a food patch), and (2) “connectedness” (the degree to which visits to food patches can be predicted from the food patches visited prior). Both measures have been applied in recursive movement analysis (Berger-Tal and Bar-David, 2015; Morrison et al., 2021). We hypothesize that revisiting at regular intervals reflects an awareness of periodic resource availability or regeneration time (e.g., ripening fruit, nectar replenishment) and that revisiting correlated with prior food patches reflects an awareness of relationships among different food resources (e.g., non-seasonal but synchronous fruiting). For example, if a primate has learned that certain fruit species emerge simultaneously, this may be reflected in a higher connectedness for food sources belonging to this species (Figure 5). These two measures can thus be used as proxies for spatiotemporal cognition; their variation with experience in each environment provides an index of the species' use of cognition in dealing with predictable variation. An analysis of the temporal pattern in which patches are visited complements our example 2 in which the spatial efficiency of a route was evaluated using network metrics. A spatial network of visits could appear as inefficient because of the temporal correlation between visited patches: two patches that are far apart could be visited in sequence precisely because of their synchronous fruiting timing.

To derive regularity and connectedness metrics from x,y,t-data, we first need to operationalize a “food patch” (e.g., a location where the animal was stationary or engaged in highly tortuous movement for a given period of time) and then represent patches and the trajectories between them as a directed graph depicting spatial (patch locations), temporal (visit order), and topological (connectedness) characteristics (Figure 5A; Pasquaretta et al., 2020). Regularity is measured by the coefficient of variation in the time it takes for a primate group to return to a patch visited before (Figure 5B). Connectedness is calculated by first establishing a frequency matrix summarizing (for all patches) the frequencies with which two patches are visited in sequence (effectively a Markov transition matrix, Figure 5C) and subsequently calculating Cramér's V for this matrix. This matrix quantifies the association between its rows (arrival patch) and columns (departure patch) (Figure 5C). The higher the association between the rows and columns, the higher the connectedness of ranging and the better one can predict the sequence in which patches are visited. In order to control for the spatial relationships between patches, which could be a simple reason why patches are visited in sequence, one can calculate the expected sequential visit matrix based on spatial proximity between patches and subsequently calculate the chi-squared statistic and Cramér's V. This estimate of Cramér's V expresses strength of association among food patches, after taking spatial proximity into account. Another way to control for spatial proximity would be to compare the observed sequences of visits to the sequence that would be obtained by visiting patches along the shortest path between patches, as in our second example. If the observed sequence is different, reflecting the species identity of synchronous species rather than the spatial relationships between patches, then again, we would have evidence that spatiotemporal cognition is playing a role in determining an animal's sequence of visits.

As with examples 1 and 2, we propose to relate these decision outputs (regularity and connectedness) to an information gradient: experience. Experience may be measured between groups—e.g., by contrasting the maximum or mean age of group members—or within groups—e.g., by changes in group composition over years. Other things being equal, positive correlations between experience and regularity or connectedness suggest learning to exploit seasonal patterns of resource availability. (In contrast, an increase in the use of sensory ability would lead to more opportunistic food patch use, rather than revisiting previously used resources at regular intervals.) The analysis would again use a hierarchical regression model design, with regularity or connectedness as a response variable, experience as a predictor variable, and group as a random effect. If food availability data are present, the connectedness model could be extended by including an interaction between experience and the synchrony level of the respective food patch species, predicting that more experienced primate groups will perform with a higher connectedness, but only for highly synchronous food species. Finally, socio-ecological descriptors could be added in a similar way as was proposed for examples 1 and 2, and, as an extension, it would also be revealing to first measure constancy and contingency of crucial resources within the home ranges for the primate groups to evaluate whether these environmental indices explain the corresponding movement-based counterparts (regularity and connectedness).

## Including ecological challenges into comparative analyses

The ultimate aim of our approach is to gain insight into the evolution and development of spatial cognition. For this, we propose to include in the comparative phylogenetic models, for each conceptual analysis, the estimates of the socio-ecological descriptors that primates face in their natural environment. Our survey revealed that currently a substantial amount of data are available to estimate ecological descriptors for many primate groups, populations, and species. Below, we discuss briefly how we can use such data to improve traditional descriptors and in what way such descriptors can best be included into our proposed models.

Several comparative phylogenetic studies have suggested that ecological complexity is an important driver of the evolution of animal cognition (Barton, 1996; Croston et al., 2015; DeCasien et al., 2017; van Woerden et al., 2010, 2012). However, when we look at specific measures of ecological challenges, these seem disappointingly simplistic: home range size, range overlap, percentage of fruit in the diet, seasonality of foods. Though readily available, these broad parameters may not be sufficient for describing the environmental complexity that a successful forager must navigate.

For instance, the positive relationship between percentage of dietary fruit and spatial memory or a relatively larger brain (Barks et al., 2015; Barton, 1996; DeCasien et al., 2017; Rosati, 2017) may imply that individuals with a larger spatial memory or hippocampus are better at finding low-density food, such as fruit, especially when locomotor costs are high (Janmaat, 2019; Janmaat et al., 2016). However, the positive relationship could equally mean that energy-rich food, such as fruit, enables larger brains and “more costly” cognition to be maintained (Barton, 1996; DeCasien et al., 2017) or that energy-poor food requires larger guts to digest and hence larger bodies, so “relatively” smaller brains may be a consequence of selection on body size (Byrne, 1996). To distinguish between these explanations, we need more detailed measures of the ecological challenges faced and more rigorous thinking about our model designs. For example, we can include the mean ephemerality levels or spatial density of the different fruit species in the diet as predictor variables, while controlling for the percentage of dietary fruit. In more than half of the studies from our survey, food availability data have been collected, making the testing of such a model a realistic possibility. Such a model could provide much more convincing evidence that brain evolution was indeed driven by the ecological challenge of finding ephemeral foods (Houle et al., 2014; Janmaat et al., 2014, 2016).

With the growing variety and amount of ecological data now being collected by field researchers, the range of complexity indicators can be extended. Specifically, we can use some of the detailed measures developed by ecologists to describe the ecological complexity of a system (reviewed in Beissinger and Gibbs, 1993; Parrott, 2010). To narrow down the number of measures, we recommend first asking what “handles” primates can use to predict the environment. For instance, since we know that the synchronous appearance of certain fruits helps monkeys to forage efficiently (de Guinea et al., 2021; Janmaat et al., 2012; Menzel, 1991), variation in fruiting synchrony could be a candidate ecological challenge. Other examples of ecological challenges that can be tackled by use of cognition are the variation in fruit production intervals between individual trees or their productivity (Janmaat et al., 2016) as well as the relative number of fruit species in a primate's home range.

Once we have selected informative socio-ecological measures, the next step is to make *a priori* predictions as to the effects they may have on cognition. Many of these effects will not be linear. For instance, home range size often shows a significant linear relationship with proxies for cognition but not always (e.g., Barton, 2000). The discovery that home range size scales with body mass according to a power function (Noonan et al., 2020) helps explain these inconsistencies. Non-linear relationships may be more general than this and may even be hump shaped. Consider the problem of locating food in an environment. As Grassberger (1986) has pointed out, the environment one would intuitively call “most complex” is neither the most ordered—imagine one where all food is at equal distance and direction of each other—nor the most disordered one. The first case is unchallenging, while the latter is near-random—chaotic and impossible to predict. In neither case would enhanced cognitive abilities improve foraging efficiency: cognition is most useful within the “zone of possibility”, where mental modeling of the environment is feasible but challenging (Riotte-Lambert and Matthiopoulos, 2020).

Apart from the need to consider non-linear effects of ecological descriptors in comparative models, it is similarly important to think about potential interaction effects of these descriptors on the evolution of cognition. For example, we might expect that the ability to plan an efficient foraging route will especially be beneficial when a primate lives in a large home range compared to a small range but only when food availability and search costs are equal for both. That is, there could be interactive effects of range size, food density, and search costs on route planning performance. In sum, we believe that there is an opportunity in future comparative analyses to (1) incorporate more detailed and informative measures of socio-ecological challenges, (2) generate specific *a priori* expectations of their effects, including non-linear effects, and (3) make predictions about the interactions of these effects.

## Conclusion

The study of primate movement data with cutting edge methods can provide important insights into the development and evolution of primate cognition. Extant ranging data from animals in natural habitats thus has considerable untapped potential. Our survey reveals that extensive ranging data, as well as corresponding socio-ecological measures, are already available for at least 164 primate populations, which creates a unique opportunity to conduct large-scale comparisons of spatiotemporal cognition and their phylogenetic and socio-ecological correlates. We have presented a conceptual framework (Figure 2) highlighting several ways in which these data can be analyzed. Many of these analyses can be conducted using only x,y,t-data; nevertheless, behaviorally annotated x,y,t-data and associated socio-ecological data would bolster the strength of these relatively simple analyses. For many study sites, such data are also available. Our aims in presenting these conceptual analyses are twofold to inspire young scholars to (1) operationalize our ideas and conduct these and similar analyses and (2) to collect comparable data on other, as yet unstudied, primate populations. The second aim is particularly urgent: the current generation of young scholars may be one of the last to be able to collect data on primates living in natural settings.
